# In Silico Approach: Anti-Tuberculosis Activity of Caespitate in the H37Rv Strain

**DOI:** 10.3390/cimb46070387

**Published:** 2024-06-27

**Authors:** Andrea Moreno-Ceballos, Norma A. Caballero, María Eugenia Castro, Jose Manuel Perez-Aguilar, Liliana Mammino, Francisco J. Melendez

**Affiliations:** 1 Laboratorio de Química Teórica, Centro de Investigación, Departamento de Fisicoquímica, Facultad de Ciencias Químicas, Benemérita Universidad Autónoma de Puebla, Edif. FCQ10, 22 Sur y San Claudio, Ciudad Universitaria, Col. San Manuel, Puebla C.P. 72570, Mexico; andrea.morenoce@alumno.buap.mx (A.M.-C.); jmanuel.perez@correo.buap.mx (J.M.P.-A.); 2 Facultad de Ciencias Biológicas, Benemérita Universidad Autónoma de Puebla, Edif. BIO1, 22 Sur y San Claudio, Ciudad Universitaria, Col. San Manuel, Puebla C.P. 72570, Mexico; 3 Centro de Química, Instituto de Ciencias, Benemérita Universidad Autónoma de Puebla, Complejo de Ciencias, ICUAP, Edif. IC10, 22 Sur y San Claudio, Ciudad Universitaria, Col. San Manuel, Puebla C.P. 72570, Mexico; mareug.castro@correo.buap.mx; 4 School of Mathematical and Natural Science, University of Venda, Thohoyandou 0950, South Africa; sasdestria@yahoo.com

**Keywords:** caespitate, antituberculosis activity, molecular docking, MM/GBSA, H37Rv strain

## Abstract

Tuberculosis is a highly lethal bacterial disease worldwide caused by *Mycobacterium tuberculosis* (*Mtb*). Caespitate is a phytochemical isolated from *Helichrysum caespititium*, a plant used in African traditional medicine that shows anti-tubercular activity, but its mode of action remains unknown. It is suggested that there are four potential targets in *Mtb*, specifically in the H37Rv strain: InhA, MabA, and UGM, enzymes involved in the formation of *Mtb*’s cell wall, and PanK, which plays a role in cell growth. Two caespitate conformational structures from DFT conformational analysis in the gas phase (GC) and in solution with DMSO (CS) were selected. Molecular docking calculations, MM/GBSA analysis, and ADME parameter evaluations were performed. The docking results suggest that CS is the preferred caespitate conformation when interacting with PanK and UGM. In both cases, the two intramolecular hydrogen bonds characteristic of caespitate’s molecular structure were maintained to achieve the most stable complexes. The MM/GBSA study confirmed that PanK/caespitate and UGM/caespitate were the most stable complexes. Caespitate showed favorable pharmacokinetic characteristics, suggesting rapid absorption, permeability, and high bioavailability. Additionally, it is proposed that caespitate may exhibit antibacterial and antimonial activity. This research lays the foundation for the design of anti-tuberculosis drugs from natural sources, especially by identifying potential drug targets in *Mtb*.

## 1. Introduction

Tuberculosis (TB) is an infectious disease caused by *Mycobacterium tuberculosis* (*Mtb*). It is one of the 10 leading causes of death worldwide [1,2], with the highest burden in Africa and Asia [3]. In Latin America, Mexico is one of the three countries with a high incidence of TB, which has the third highest estimated burden of drug-resistant tuberculosis (DR-TB) and multidrug-resistant tuberculosis (MDR-TB), with 4007 reported cases in 2020 [4]. In recent years, one of the main problems has been the development of multidrug resistance by *Mtb* [5,6,7]. It makes the necessary identification and development of new molecular structures more efficient in the treatment of this disease.

Natural products represent a vast source of molecular structures with interesting biological activities, such as antigonorrhea, antifungal, and antioxidant; and to treat respiratory infections, such as pneumonia, sinuses, and tuberculosis [8,9,10,11,12]. In several countries, a portion of the population uses plants to combat various health issues and improve the quality of life. *Helichrysum caespititium* is a plant used in traditional South African medicine that contains a large quantity of acylphloroglucinols, including the caespitate molecule, which exhibits anti-tuberculosis activity [12,13].

Acylphloroglucinols (ACPLs, [14]) are a class of compounds that are structurally derived from 1,3,5-trihydroxybenzene and have at least one acyl group (R-C=O). Most of them show biological activity and are considered potential compounds for treating various diseases [15]. Most ACPLs are characterized by the presence of one or more intramolecular hydrogen bonds (IHBs) [16,17,18,19,20,21,22,23].

**Figure 1:**
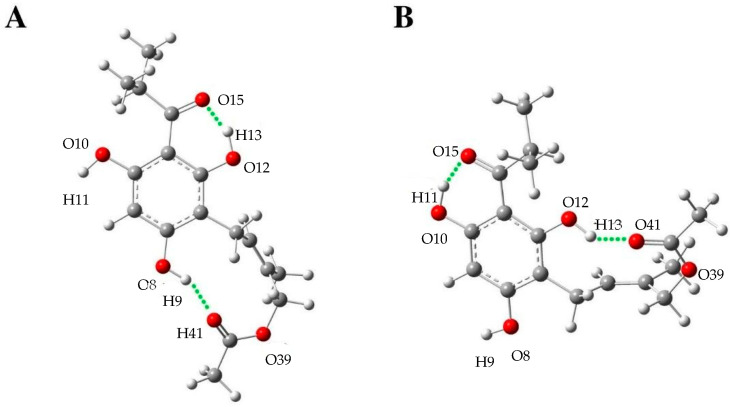
Intramolecular hydrogen bonds (highlighted with green lines) identified in the optimized molecular structure of the caespitate conformers used in this work. (**A**) CG, extended conformation preferred in the gas phase. The two IHBs identified in the global minimum are formed by the O12−H13⋯O15 atoms and by the O8H9⋯O41 atoms. (**B**) CS, hairpin conformation favored in the DMSO solution phase. Similar to the CG conformation, two IHBs identified in the global minimum are formed by the O10−H11⋯O15 atoms and by the O12−H13⋯O41 atoms.

In the caespitate molecule (Figure 1), the *sp*^2^ oxygen atom of the acyl chain (O15) can form an IHB with one of the two –OH groups in the *ortho* position of the ring, i.e., with H11 or H13; this IHB is referred to here as the first IHB. In addition, a second IHB can be formed between H9 or H13 and one of the O atoms of the ester group in the prenyl chain, where O41 or O39 are the acceptors that are stronger [17]. Moreover, the double bond of this chain leads to the formation of *Z* and *E* isomers, with the former being able to inhibit the growth of drug-resistant strains of *Mtb* [19,20,21,22,23].

The therapeutic potency of caespitate has been investigated over the last few years. In vitro studies with DMSO (1%) in the medium to not affect bacterial growth have shown that caespitate has synergistic effects with anti-tuberculosis drugs such as isoniazid, rifampicin, ethambutol, streptomycin, and ethionamide. This phloroglucinol has been evaluated for its mycobacterial activity in drug-resistant and drug-sensitive (H37Rv) *Mtb* strains and showed a Minimum Inhibitory Concentration (MIC) value of 0.1 mg/mL. This prompted us to investigate the possible biological receptors or targets that caespitate could couple with to achieve the biological activity shown in the experiments [23].

Several enzymes involved in key processes for *Mtb* survival have become attractive anti-TB targets due to their physiological function or absence in mammals [24,25].

In this regard, molecular docking and MM/GBSA studies are performed to predict the interactions between the caespitate molecule and three essential enzymes involved in *Mtb* cell wall biosynthesis, namely 2-trans-enoyl-ACP reductase (InhA), β-ketoacyl-ACP reductase (MabA), 5′-diphosphate (UDP) galactopyranose mutase (UGM), and the last one, pantothenate kinase (PanK), the first enzyme of CoA biosynthesis.

InhA and MabA enzymes share structural and functional similarities, as their main functions are based on the production of very-long-chain fatty acid derivatives, which are important precursors of the main lipids of the mycobacterial envelope [26]. Moreover, they are similarly inhibited by the first-line anti-tuberculosis drug isoniazid [26,27,28].

On the other hand, the flavoenzyme UGM is an essential biocatalyst involved in the growth of some pathogenic microorganisms, including *Mtb*. This protein catalyzes the conversion of uridine diphosphogalactopyranose (UDP-GalP) via the 2-keto intermediate into uridine diphosphogalactofuranose (UDP-GalF), which is an important building block for the construction of the *Mtb* cell wall [29,30,31,32,33,34].

**Scheme 1:**
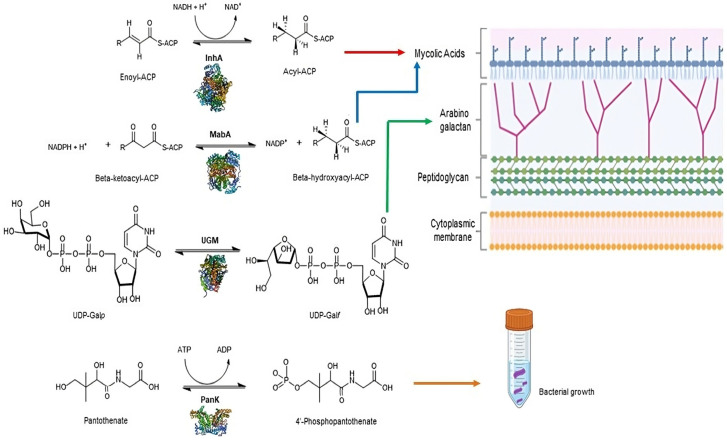
Chemical reactions catalyzed by the four enzymes proposed with distinct localization: those involving the InhA and MabA enzymes take place within the cell wall of *Mtb*, specifically in the mycolic acids (highlighted with red and blue arrows), while the reaction catalyzed by UGM is situated in the arabinogalactans (highlighted with a green arrow). Additionally, the reaction mediated by PanK contributes to the bacterium’s growth process (highlighted with a yellow arrow).

Finally, the PanK type I enzyme catalyzes the phosphorylation of pantothenate (vitamin B5) to 4′-phosphopantothenate in the first and rate-limiting step of the coenzyme A (CoA) biosynthetic pathway [35]. CoA is an essential cofactor for the regulation of enzymes involved in numerous cellular metabolic pathways, such as lipid biosynthesis [36]. For these important reasons, InhA, MabA, UGM, and PanK are attractive pharmacological targets (Scheme 1).

Computational methods are valuable tools that provide insight into how a molecule (ligand) can bind to the active site of a protein (receptor) to block or promote its function [37], which has yielded data that are consistent and complementary with results from experimental biology [38]. The aim of this research is to perform molecular docking studies to predict the possible molecular mechanism by which the caespitate molecule elicits its anti-tuberculosis activity. Four essential enzymes, namely InhA, MabA, UGM, and PanK that are present in the well-studied H37Rv *Mycobacterium tuberculosis* strain, are proposed as possible therapeutic targets. The Molecular Mechanics/Generalized Born Surface Area (prime MM/GBSA) method is used to improve the docking scores and predict the total binding energies [39].

## 2. Materials and Methods

### 2.1. Protein, Ligand, and Grid Box Preparation

The most populated and energy-stable conformers of caespitate, which were previously optimized using the APFD functional and 6-311+G(2d,p) basis sets, in gas (CG) and solution with DMSO (CS) phases [40], were used as ligands for molecular docking calculations. The solvent effect of DMSO was taken into account with the Solvation Model based on Density (SMD) method [41].

The validation of the methodology was performed with the isonicotinic-acyl-NADH for the MabA enzyme, which was obtained from the crystal structure (PDB:1ZID). This molecule was used as a control inhibitor in previous work [42]. On the other hand, for the InhA, UGM, and PanK enzymes, a re-docking trial was performed, looking for alignment of the docked positions obtained with those of the co-crystallized structure. The RMSD values (Å) between the native ligands and the re-docked poses had values of less than 1 Å for InhA, UGM, and PanK. The crystal structures of InhA (PBD:1BVR), MabA (PDB:1UZN), UGM (PDB:4RPJ), and PanK (PDB:4BFW) were retrieved from the Protein Data Bank database (https://www.rcsb.org/, accessed on 30 May 2024). Water molecules and non-standard residues were removed with UCSF Chimera software, 1.17.1 [43]. AutoDock Tools (ADT) was crucial to adding polar hydrogens and Kollman charges for the protein, while Gasteiger charges were added for the ligand. All rotatable bonds detected by the program were treated as non-rotatable. The size of the search box was set as 30 × 30 × 30 Å for all proteins, except for MabA, which was 22 × 22 × 22 Å. The center for each grid box was set as x = 17.067, y = 15.545, and z = 8.741, x = 27.707, y = −2.400, and z = 18.747, x = −18.591, y = −9.146, and z = 11.124, and, x = 3.561, y = 17.242, and z = 11.951 Cartesian coordinates for InhA, UGM, PanK, and MabA, respectively. The parameters for MabA were taken from a previous study [42], as these sizes include the residues of the enzyme binding site. The molecular docking calculations were performed using AutoDockVina 1.1.2 software [44], which uses the genetic Lamarckian algorithm that modifies the geometry of the ligand to match the pocket of the protein it interacts with (the pocket itself is not changed) [45]. The active site for the four enzymes has been identified and reported in previous research [27,46,47,48]. In addition to selecting therapeutic targets for their important physiological function or absence in humans, multiple sequence alignment was performed using Clustal Omega tool 1.2.4 (https://www.ebi.ac.uk/jdispatcher/msa/clustalo, accessed on 5 June 2024) software. This multiple alignment was performed between InhA, MabA, UGM, and PanK proteins. The results showed that there is no relevant similarity or functional or evolutionary relationship between the selected genes or proteins; see Figure S1.

On the other hand, a structural alignment was carried out with the PDBeFold tool (www.ebi.ac.uk/msd-srv/ssm/, accessed on 5 June 2024), which allows the identification of structural similarity of secondary structures between proteins. This structural alignment was performed between the four proteins. To ensure that the selected targets were structurally distinct, a structural alignment was performed, obtaining RMSD values between 2.06 and 5.18 Å. However, an analysis of the 3D structures revealed that there is no alignment of the alpha- and beta-folded secondary structures in all enzymes except MabA and InhA; see Figure S2.

The Glide program by Schrödinger Suite software 2018-4 [49,50,51] was used to perform the same docking calculations carried out with AutoDock Vina 1.5.7 software. The structures of the ligands and the enzymes were prepared with the default steps of the program. Water molecules and non-standard residues were removed using the Protein Preparation Wizard, and the corresponding partial charges were assigned. The grid was set at the active site of each enzyme. Rigid docking was carried out, and the best pose was selected based on the Glide XP docking score. These results were used for MM/GBSA calculations.

### 2.2. MM/GBSA Calculations

The Molecular Mechanics/Generalized Born Surface Analysis (MM/GBSA) method arose as a result of the development of more accurate methods than molecular docking to predict the affinity of a ligand for a receptor, which has been used to improve the results of docking and reproduce experimental data. The ligand pose structures obtained from AutoDock Vina and Glide docking algorithms were used as the starting point to perform the simulation. The relative binding free energy was calculated using the following equation:

(1) ΔG(bind) = ΔG(solv) + ΔE(MM) + ΔG(SA)

where ΔG(solv) is the difference in GBSA solvation energy of the protein–CS complex and the sum of the solvation energies for the unliganded protein and CS; ΔE(MM) is a difference in the minimized energies between the protein–CS complex and the sum of the energies of the unliganded protein and CS; and finally, ΔG(SA) is a difference in the surface area energies of the complex and the sum of the surface area energies for the unliganded protein and CS. Prime MM/GBSA [52] calculates the energy from the minimization of the complex, free protein, and free CS using the force field OPLS_2005. The CS strain energy is also calculated using the protocol with the VSGB solvation model.

### 2.3. Analysis of Pharmacokinetic Parameters (ADME and Ames)

To assess the potential of caespitate as a promising pharmaceutical candidate, an ADME (absorption, distribution, metabolism, and excretion) study was performed using the QikProp module [53] within Schrödinger suite 2018-4. QikProp employs a structural analysis to generate physical descriptors, considering atoms and their charges, as well as the volume and surface of the molecule. This approach enables accurate predictions of the pertinent pharmacological properties linked to ADME characteristics. Furthermore, QikProp efficiently evaluates the ADME properties of the chosen molecules, adhering to Lipinski’s Rule of Five (RO5) [54]. On the other hand, the Ames test was carried out with AdmeSAR 2.0 [55] prediction software, which provides information about drug toxicity. The SMILES of completed ligands were entered into the program to assess their toxicity, specifically concerning the Ames test [56] prediction to verify whether the compound could induce DNA changes.

## 3. Results

### 3.1. Molecular Docking Calculations

Molecular docking is a widely used computational tool to find the main interaction sites by evaluating the different poses with their respective binding affinity energies. Therefore, an in silico study to discover the way in which the caespitate molecule induces its anti-tuberculosis activity is presented. Previously, our group investigated the conformational space explored by caespitate in the gas phase (CG, extended conformation, Figure 1A) and in solvent conditions (CS, hairpin conformation, Figure 1B). In the global minimum of electronic energy, both conformers form two intramolecular hydrogen bonds (IHBs). In the CG conformation, the IHBs are formed by the O12−H13⋯O15 atoms and by the O8−H9⋯O41 atoms, whereas in the CS conformation, the IHBs are formed by the O10−H11⋯O15 atoms and by the O12−H13⋯O41 atoms (see the green lines in Figure 1A,B). Several studies have reported that IHBs are an important interaction with a crucial role in the modulation of biological and chemical properties, e.g., IHBs are involved in the modulation process of inhibitor recognition by enzymes [57,58], in profoundly influencing the detoxification pathways in the liver [22], and in impacting the mechanism of action between organometallic drugs and receptor proteins [59]. In this sense, it would be desirable to know whether the previously characterized IHBs are relevant for the biological activity of caespitate. The characterization of the IHBs in caespitate was previously studied in detail using the c-DFT and QTAIM approaches [40].

**Figure 2:**
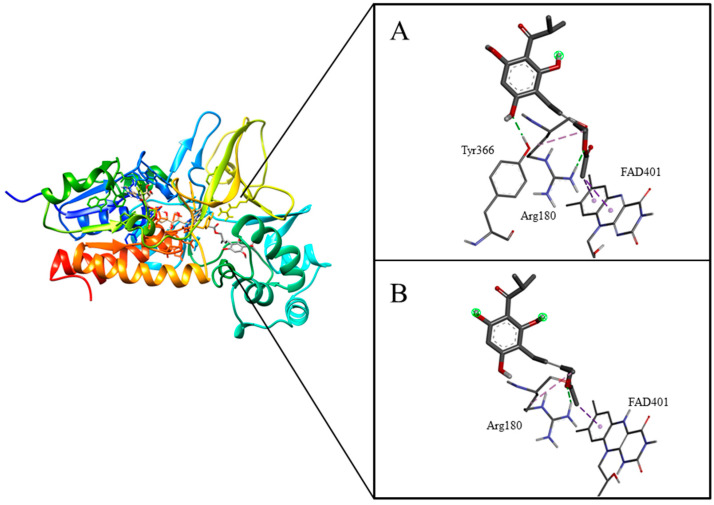
(**A**) Graphical 3D representation of the main interactions in the UGM–CS complex after semiflexible docking simulation in Glide. (**B**) Graphical 3D representation of the main interactions in the UGM–CS complex after semiflexible docking simulation in AutoDock Vina.

**Figure 3:**
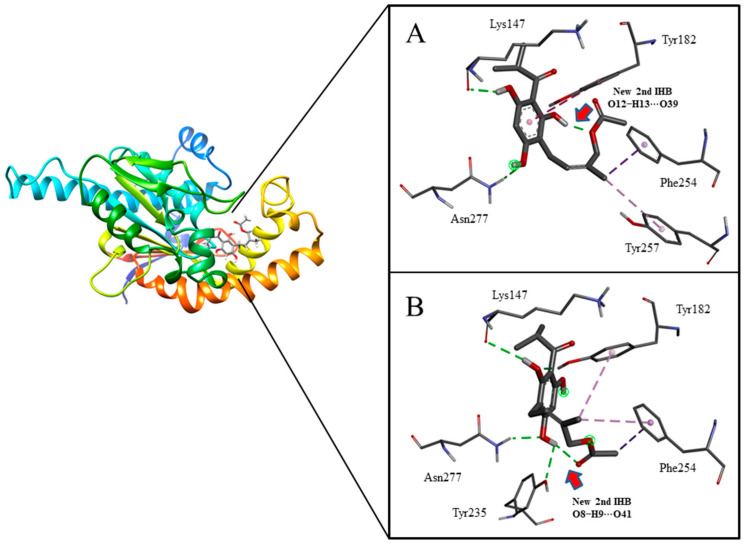
(**A**) Graphical 3D representation of the main interactions in the PanK–CS complex after semiflexible docking simulation in Glide. (**B**) Graphical 3D representation of the main interactions in the PanK–CS complex after semiflexible docking simulation in AutoDock Vina.

**Figure 4:**
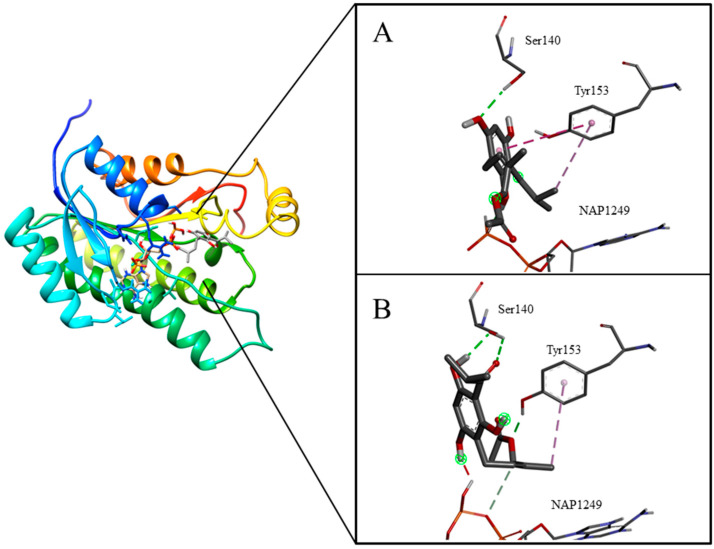
(**A**) Graphical 3D representation of the main interactions in the MabA–CS complex after semiflexible docking simulation in Glide. (**B**) Graphical 3D representation of the main interactions in the MabA–CS complex after semiflexible docking simulation in AutoDock Vina.

**Figure 5:**
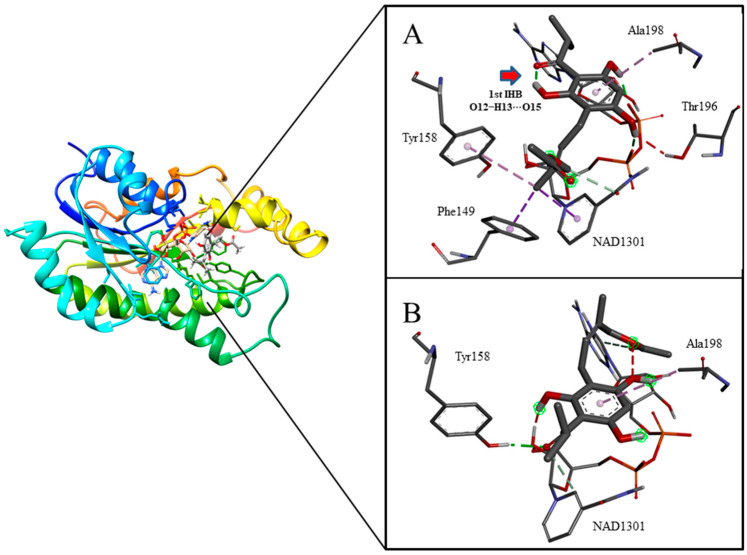
(**A**) Graphical 3D representation of the main interactions in the InhA–CS complex after semiflexible docking simulation in Glide. (**B**) Graphical 3D representation of the main interactions in the InhA–CS complex after semiflexible docking simulation in AutoDock Vina.

Semi-flexible docking. Calculations of semi-flexible docking, i.e., where the ligand bonds are free to rotate while the protein bonds remain rigid, were performed using the Glide and AutoDock Vina programs. Notably, three of the enzymes contain a cofactor that aids in the catalysis and was included during the docking calculations (InhA contains NAD, MabA contains NAP, and UGM contains the FAD cofactor). The results from the Glide program indicate that both CG and CS conformers converge to the same molecular pose. Additionally, the analysis shows that the caespitate complexes formed with UGM and PanK have the best affinity energies with values of −9.2 and −8.0 kcal mol^−1^, respectively; see Table 1. In UGM, an alkyl and a hydrogen bond interaction with the Arg180 residue are detected; see Figure 2A. A hydrogen bond interaction with the Tyr366 residue and a π–σ interaction with the cofactor FAD are observed. Moreover, none of the complexes retained any hydrogen bonds. For PanK, Figure 3A, a π–π interaction with the amino acid Tyr182, a π–σ interaction with the residue Phe254, and a hydrogen bond interaction with the residue Asn277 are noted. The conservation of the second IHB, O12−H13⋯O39, is observed in the caespitate ligand. For MabA, Figure 4A, hydrogen bond interactions with the residue Ser140 and the cofactor NAP are observed via π–π and π–alkyl interactions. On the other hand, no IHBs are conserved. For the enzyme InhA, the principal interactions are π–alkyl type with the amino acids Ala198, Tyr158, and Phe149. An interaction via a hydrogen bond and a C−H bond with the cofactor NAD is observed. However, an unfavorable donor–donor interaction with the amino acid Thr196, located in the substrate-binding loop region, is observed. Finally, it is noteworthy that the caespitate ligand retains the first IHB, O12−H13⋯O15; see Figure 5A.

**Figure 6:**
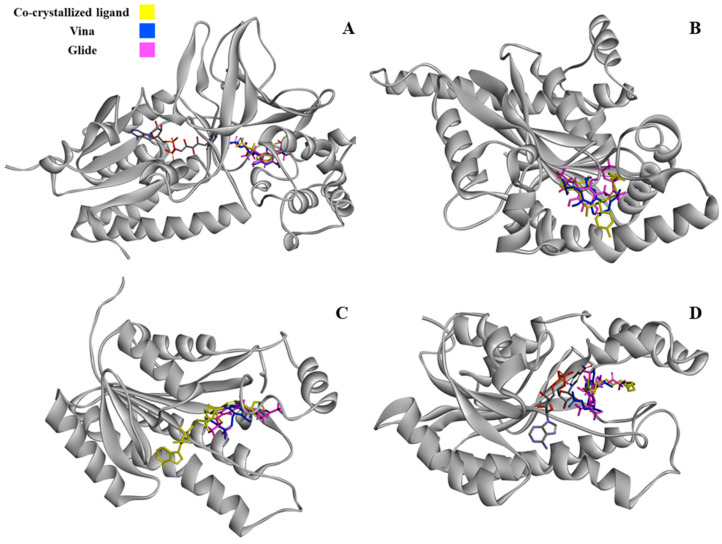
Graphical 3D representation of the initial complex models with the complexes obtained in Vina and Glide. (**A**) UGM enzyme; (**B**) PanK enzyme; (**C**) MabA enzyme; and (**D**) InhA enzyme.

Furthermore, the results of semi-flexible docking in *AutoDock Vina* indicate that regardless of the initial conformation, CG or CS, the resulting molecular poses of caespitate are very similar, and consequently, its Vina score value (Table 1). In particular, according to Vina, the UGM/caespitate complexes, using the CG and CS conformations as the starting ligand structures, show the values of −8.7 and −8.8 kcal mol^−1^ for the binding affinities, respectively (Table 1). As for the case of the PanK enzyme, which does not contain any cofactor in its structure, the resulting values for the binding affinities are −7.3 kcal mol^−1^ (CG) and −7.0 kcal mol^−1^ (CS), respectively (Table 1). In the case of the other two systems, MabA–CS (Figure 4B) and InhA–CS (Figure 5B) complexes, their binding affinity values are below of previous ones (Table 1), and unfavorable donor–donor interactions with the cofactors NAP and NAD were observed, respectively; see Table S1. Notably, neither of the two original IHBs in CG and CS are retained. Interestingly, the best molecular pose from the docking calculations that utilized the CS conformation of the ligand forming a complex with the PanK enzyme displays a new IHB formed by the O8−H9⋯O41 atoms; see Figure 3B. The interactions obtained in AutoDock Vina for the enzyme–caespitate complexes are elucidated below. In UGM, both CS (Figure 2B) and CG (Figure S3) conformers show an interaction with the amino acid Arg180 via a hydrogen bond. Furthermore, another interaction is observed between the same residue and the oxygen atom of the ester group of the prenylated chain of caespitate–CS complexes. Also, the molecular pose shows a hydrogen bond interaction that CG forms between the O atom of the carbonyl group in the prenylated chain and the hydroxyl group of residue Tyr366 (Figure S3B). Furthermore, CS (Figure 2B) and CG (Figure S3B) show a π–σ interaction with the FAD cofactor. Concerning IHBs, the conformers do not maintain any such bonds. In Figure 3B, the interactions from the semiflexible docking of the CS–PanK complex are depicted. In the PanK–CS (Figure 3B) and PanK–CG (Figure S4) complexes, hydrogen bond interactions with residue Tyr235 are observed. Moreover, in the PanK–CS complex, π–σ and π–alkyl interactions with the residue Phe254 are also observed (Figure 3B). Additionally, in the PanK–CS complex, interactions via hydrogen bonds and π–alkyl with the Tyr182 residue and hydrogen bonds with Asn277 are observed (Figure 3B). Regarding the conservation of IHBs, CG does not maintain any IHBs, whereas in CS, a new IHB, O8−H9···O41, is formed (Figure 3B). In Figure 4B, the interactions from the semiflexible docking of the complexes with the enzyme MabA–CS are depicted. In the MabA–CG (Figure S5B) and MabA–CS (Figure 4B) complexes, an interaction via a hydrogen bond with the residue Tyr153 is observed, and CS also shows a π–alkyl interaction with the same residue (Figure 4B). In MabA–CS, hydrogen bond interactions with the residue Ser140 are observed. An interaction with the cofactor NAP via a C−H bond and an unfavorable donor–donor interaction are noted. Additionally, the ligands CG and CS do not retain any intramolecular hydrogen bonds in their structure. In Figure 5B, the interactions from the semiflexible docking of the InhA–CS complex are depicted. The InhA–CS complex exhibits interactions with NAD in the form of a C−H bond and an unfavorable donor–donor interaction, and a π–alkyl interaction with Ala198 is established (Figure 5B). A hydrogen bond interaction with the residue Tyr158 is observed. In the InhA–CG complexes (Figure S6), a hydrogen bond interaction with NAD and the residue Ala198 are observed (Figure S6B). Finally, it is noted that neither CG nor CS retain any intramolecular IHBs. After obtaining the results, a comparison was made between the enzyme complexes and the systems obtained in both Glide and AutoDock Vina. It is clearly evident that caespitate interacts in the active site of all four enzymes; see Figure 6.

Rigid docking. To understand the possible role of the IHBs, we decided to perform rigid docking calculations (both protein and ligand bonds remain rigid), where the original interactions identified previously are retained. As expected, the values of Glide are worse when using the caespitate rigid structure; however, the results from AutoDock Vina were unexpected because they showed better results for all the protein complexes in the CG conformation. Additionally, the AutoDock Vina score shows the same trend for this case (CG), with the UGM complex displaying the best value, followed by PanK, MabA, and InhA (Table 2). The CG–enzyme complexes results will be discussed here, whereas the CS–complexes results are included in the Supplementary Materials section, see Figures S11–S14.

For UGM (an enzyme with different functionality), the affinity energy value in AutoDock Vina for an UGM–CG complex was smaller by more than four of kcal mol^−1^ with respect to the common substrate–UGM complex (−13.6 kcal mol^−1^) (Table S2). Table 3 and Figure S7B show the main interactions of the UGM–CG complex, highlighting the interactions with the residues Arg180 via a π–donor hydrogen bond and Tyr366 via a π–alkyl bond. In Table 2, the Glide score is presented. The UGM–CG complex displays a docking score value of −5.7 kcal mol^−1^. The interactions are depicted in Figure S7A. The interactions between CG and amino acid Tyr366 via π–π stacking and π–alkyl are observed, as well as with the amino acid Arg180 via van der Waals forces.

The PanK enzyme was proposed to be analyzed because it is involved in an essential pathway for the growth of *Mtb*. The AutoDock Vina score for caespitate–CG and PanK was –9.0 kcal mol^−1^ (Table 2). The docking results for CG are displayed in Figure S8B, where an interaction with Phe254 via π–alkyl is presented. Additionally, an interaction via a hydrogen bond with the residue Tyr257 is observed, along with a π–π interaction with the amino acid Tyr182. The Glide score is presented in Table 2, where the PanK–CG complex has a Glide score of −7.2 kcal mol^−1^, representing the best docking score among the systems calculated in this program using the rigid docking protocol. Figure S8A shows the interaction between CG and Phe254 via van der Waals forces, with Tyr257 via hydrogen bonding, as well as with Tyr235 via π–alkyl interactions, and establishes a hydrogen bond interaction with Tyr182 and with Asn277 via van der Waals forces.

The MabA enzyme shares similar functionality to the InhA enzyme. The Vina docking score value observed for the MabA–CG complex (Table 2) as for the InhA–CG complex was smaller by two kcal mol^−1^ than the control inhibitor of MabA (isonicotinic-acyl-NADH), whose score value was −9.5 kcal mol^−1^ [42]. We observed that CG forms a hydrogen bond between the O atom of the carbonyl group of the prenylated chain and the H atom of the side chain of the Tyr153 residue (Figure S9B). Table 2 displays the Glide scores, the MabA–CG complex has a docking score of −5.78 kcal mol^−1^. Figure S9A presents van der Waals interactions between CG and the amino acids Tyr153, Ser140, and Lys157 and the cofactor NAP.

For the InhA–CG complex, the Vina binding affinity value (Table 2) was smaller in more than two kcal mol^−1^ to the common substrate–InhA complex (−9.5 kcal mol^−1^) (Table S2). On the other hand, Figure S10B displays an interaction between CG and the cofactor NAD via a hydrogen bond. The InhA–CG complex shows a binding affinity value of −5.5 kcal mol^−1^. Figure S10A presents the interactions between CG and the amino acid Arg254 via van der Waals forces and shows an interaction with the cofactor NAD via hydrogen bonding.

### 3.2. MM/GBSA Calculations

The prime MM/GBSA method is more theoretically rigorous than the docking one, which uses a scoring function based on the docking complex. Prime MM/GBSA provides an accurate prediction of the ligand–protein interaction, and in this study, it was used based on the docking complex to improve the score values of the calculations from both the AutoDock Vina and Glide programs. According to AutoDock Vina, the best molecular poses were obtained via a rigid docking methodology, starting from the UGM-CG (−9.3 kcal mol^−1^) and PanK-CS (−9.0 kcal mol^−1^) systems. On the other hand, Glide showed that the best molecular poses were obtained from the UGM/semi-flexible protocol. In consequence, those poses are employed to perform the MM/GBSA methodology. Regarding the MM/GBSA results, it is observed that the UGM/caespitate complex shows a strong binding energy (−84.5 kcal mol^−1^) compared to other complexes. UGM/caespitate (−9.2 kcal mol^−1^) and PanK/caespitate (−8.0 kcal mol^−1^) systems obtained using this trend are further supported by docking analysis, where complexes formed with PanK show similar stability, ranking second in terms of stability. The Prime MM/GBSA method utilizes an additive approach, where the total binding free energy is calculated as the sum of individual energies, such as the Coulomb energy and binding free energy (NS). In this context, the contribution of Coulomb energy to the total binding energy and binding free energy (NS) in the results are remarkable (Table 4). Specifically, the MM/GBSA ΔG Bind (NS) energy is calculated using the following equation: Complex − Receptor (from the optimized complex) − Ligand (from the optimized complex), where NS represents no strain, indicating the binding/interaction energy prior to accounting for the conformational changes necessary for the formation of the complex between the receptor and the ligand.

The MM/GBSA energy parameters of each caespitate–enzyme complex are shown in Table 5. ΔG bind Lipo and ΔG bind vdW energies are the most important contributions for determining the average free energy of binding for all complexes. In particular, UGM/caespitate and Pank/caespitate systems obtained their stabilization by ΔG bind vdW and ΔG bind Lipo contributions, but ΔG bind Hbond is the lowest contributor, indicating a poor stabilization of the hydrogen bonds between caespitate and amino acids residues of PanK; in contrast, UGM/caespitate had the stabilization by ΔG bind Hbond contribution compared to the other complexes. The ΔG bind vdW term reveals a good contact between the active sites of both enzymes with the caespitate ligand. On the other hand, the ΔG bind Lipo contribution shows that the binding sites of UGM and PanK are lipophilic and, as consequence, the lipophilic interactions are dominant.

### 3.3. ADME and Ames

The ADME study provides crucial information on how a compound behaves in the body once administered. These parameters are critical in determining the efficacy and safety of a compound in drug design. This study was performed on both CG and CS caespitate conformers to evaluate their properties. The results obtained (Table 6) indicate that there are no violations of the Lipinski rule, which is a marker for assessing solubility, absorption, bioavailability, and permeability for both conformers [54,60,61]. The HB donor and HB acceptor parameters had values of 1 and 4.3, respectively, indicating the ability of caespitate to form hydrogen bonds with specific biological receptors or other biological molecules possibly influencing its pharmacological activity, absorption, distribution, metabolism, and excretion in the body. The values for the octanol/water partition parameter (QPlogPo/w) fall within the recommended range and are considered acceptable. This parameter is a measure of the lipophilicity of a compound, that is, its relative affinity for water and for octanol, which is a lipophilic molecule that is used as a simplified model of biological membranes. On the other hand, seven metabolic sites were found where oxidation or reduction reactions can be carried out mainly by enzymes in the liver and other tissues. The percentage of oral absorption is very close to one hundred percent, which suggests a good ability of the compound to cross intestinal barriers and be absorbed into the bloodstream. The logarithm values of the brain/blood partition (QPlogBB) are negative, indicating that the compound has a greater affinity for the blood than for the brain. On the other hand, the solvent-accessible surface area (SASA) values indicate that the molecule is folded or compact, with less area exposed to the solvent. Finally, the Ames toxicity study shows that caespitate is a non-toxic compound, suggesting that this molecule may not induce mutagenicity. In addition, its values (see Table 6) are within acceptable ranges for the inhibition of the inwardly rectifying potassium channel (hERG), which is associated with possible adverse cardiovascular effects.

## 4. Discussion

The semiflexible and rigid molecular docking methodology using the Glide and AutoDock Vina algorithms shows significant interactions with amino acids that play crucial roles in the four enzymes studied. In UGM–caespitate complexes, CG and CS displayed interactions with Arg180, a key residue for UGM catalysis [46], and Tyr366, known for its pivotal role in stabilizing the β-phosphate group of the UDP substrate [62]. The caespitate–PanK complexes show interactions with Tyr182 and Phe254; both residues are part of the preformed tunnel in the enzyme’s active site, essential for its normal function [47,63]. Additionally, an interaction with the Asn277 residue, involved in binding pantothenate and phosphopantothenate—precursors of CoA—was observed [47,64,65]. Furthermore, interactions with Tyr235 are found, which is necessary for binding the intermediates pantothenate and phosphopantothenate. On the other hand, it is noteworthy to highlight the significance of the interaction with the Ser140 residue in MabA complexes, given its important role as part of the catalytic triad (Tyr153, Ser140, and Lys157) of the MabA enzyme [66]. Additionally, the interaction with Tyr153, another member of the catalytic triad, is crucial for the enzyme’s acid–base catalysis [67]. These residues, along with Lys157, constitute the catalytic triad and are integral to the acid–base catalysis processes of the MabA enzyme [66]. In the InhA enzyme, an unfavorable donor–donor interaction is observed with the amino acid Thr196 in the region of the substrate-binding loop, contrary to the situation observed with potent inhibitors of this enzyme, where they have been reported to establish a favorable hydrogen bonding interaction [67]. Moreover, an unfavorable donor–donor interaction with NAD and an interaction with Ala198 are established. This residue helps stabilize intermediates and is part of the substrate-binding loop located within the enzyme’s active site [48]. Surprisingly, it was observed with the rigid docking calculation that although the complex formed by UGM and caespitate obtained a lower value than that presented in the UGM–common substrate complex, it was higher than that obtained in the UGM–psoromic acid complex (−7.4 kcal mol^−1^) [68], which has been reported to be a good experimental inhibitor (Table 2). Notably, in the Glide results, this complex exhibited a significantly lower energy value in kcal mol^−1^ compared to both the common substrate and the aforementioned inhibitor. Contrary to our expectations, the Vina binding affinity value for the InhA–CG complex was lower than the common substrate, eliminating the theory that caespitate might exhibit inhibitory activity on this target protein.

On the other hand, the Glide algorithm (Table 2) indicates that caespitate displays the best affinities for the UGM and PanK enzymes, regardless of the initial conformations utilized (CG or CS). In the case of the AutoDock Vina methodology, the program proposed the same trend as Glide when the CG conformation is used as the initial conformation for caespitate; that is, UGM presents the best affinity, followed by PanK, MabA, and InhA. Although UGM and InhA are identified as the best and worst complexes when using the CS conformation for caespitate, according to the AutoDock Vina score, the other two proteins do not show the same trend as Glide. Following the proposition of the relevance of the two IHBs formed in the initial structures, rigid docking methodologies were performed to retain these important features [17]. As expected, the lack of flexibility in the ligand produces worse affinities in Glide; however, and unexpectedly, the score in AutoDock Vina showed improved affinities for all the complexes (Table 1 and Table 2).

In the PanK enzyme, the second intramolecular hydrogen bond (IHB), O12−H13⋯O39, is conserved when using the Glide algorithm, whereas in AutoDock Vina, a new second IHB, O8−H9⋯O41, is formed. In the InhA–caespitate complex, the first intramolecular hydrogen bond (IHB), O12−H13⋯O15, is conserved in Glide (see Figure 5A). Therefore, the formation or conservation of the second IHB involving the prenylated chain could play a crucial role in stabilizing the PanK–caespitate complex, making it one of the complexes with the highest affinity energy.

After these findings, we chose to enhance and verify whether caespitate displayed a clear preference for UGM and PanK by calculating its corresponding free energies with the MM/GBSA method. Based on these results, we can infer that caespitate has a better affinity for these enzymes with possible interesting biological effects, which could be due by disrupting catalysis of one step in the CoA biosynthetic pathway. Furthermore, we could deduce that caespitate had a better affinity for the enzymes UGM and PanK, with interesting biological effects. When PanK is present in specific bacteria such as *Escherichia coli* [47] and *Brevibacterium ammoniagenes* [69], an antibacterial action can be attributed. On the other hand, UGM is not found in mammals and has been characterized in other important human pathogens such as *Leishmania major* and *Klebsiella pneumoniae* [70,71].

The results derived from the ADME methodology suggest that caespitate exhibits a good ability to cross biological membranes and be absorbed in the gastrointestinal tract. In addition, it has hydrogen bond acceptor sites and has OH groups that act as hydrogen bond donors, influencing its ability to establish specific interactions with other molecules or proteins in the organism. The limited exposure to the solvent indicates a lower solubility in water, but this does not seem to affect its absorption and distribution capacity since it has a notable affinity for the lipid phase. Furthermore, caespitate has favorable pharmacokinetic properties for oral absorption and could be metabolized in the body without exhibiting effects on the central nervous system.

## 5. Conclusions

Tuberculosis is an outstanding health problem worldwide due to its drug resistance. Therefore, the search for new specific compounds to treat this disease has become imperative. In this study, four essential enzymes as possible targets for caespitate are proposed, InhA, MabA, UGM, and PanK, identified in H37Rv, the most studied strain of *Mtb*. Caespitate is a phytochemical compound with anti-tuberculosis activity but with unknown mechanism of action. Molecular semi-flexible and rigid docking studies were performed, revealing interactions of caespitate with key amino acids in both methodologies. Furthermore, the results suggest that the presence of the IHBs in caespitate may not be essential for biological activity, as the interaction in the binding pocket is not conditioned by the conservation of these IHBs in both ligands. Docking calculations were not able to describe the stability to keep both H-bonds or one of them; as a result, the next step involves molecular dynamics calculations to assess and establish their stability. In addition to these limitations, this methodology pointed to the UGM and PanK enzymes as the best possible targets to caespitate with the formation of the most stable complexes. It was confirmed with the MM/GBSA-ΔG-Bind energy calculations based on the docking complex, where the ΔG bind vdW and ΔG bind Lipo parameters were the most contributors to average free energy. It is important to highlight that the MM/GBSA approach was key in this study by enhancing the accuracy of binding energy for the well-established molecular docking complexes. Therefore, it could be inferred that caespitate possibly has varying biological effects by different mechanisms of action. The anti-tuberculosis effect could be by two ways: (1) inhibiting the enzymatic action of PanK blocking the synthesis of coenzyme A (CoA), which plays a crucial role as a carrier of acyl groups and is vital for the processes of respiration and lipid metabolism in *Mtb*; and (2) inhibiting the catalytic function of UGM by blocking the interconversion between UDP-galactopyranose (UDP-GalP) and UDP-galactofuranose (UDP-GalF), a critical step in the formation of the *Mtb* cell wall. On the other hand, since both enzymes are not exclusive to *Mtb*, caespitate could have antibacterial and parasitic activities. The pharmacokinetic parameters suggest that caespitate has promising pharmacological potential due to its high likelihood of crossing biological barriers and its propensity for metabolism. Finally, these results provide a promising perspective in the realm of anti-TB drug development, so that in the future, experimental studies could be performed only with the selected enzymes from previous studies such as the present one. Finally, caespitate can be used as a template for analogues and the development of potent *Mtb* enzyme inhibitors.

## Figures and Tables

**Table 1:** Binding energies, in kcal mol^−1^, of semiflexible docking of CS enzyme and CG enzyme complexes calculated with different methodologies.

Enzyme	Glide Score		Vina Score	
	CG	CS	CG	CS
UGM	−9.2	−9.2	−8.7	−8.8
PanK	−8.0	−8.0	−7.3	−7.0
MabA	−6.7	−6.7	−7.0	−7.2
InhA	−6.3	−6.3	−5.9	−6.5

Vina Score = binding energy calculated in AutoDock Vina; Glide score = binding energy estimation in Glide.

**Table 2:** Binding free energies, in kcal mol^−1^, of the rigid docking of CG enzymes complexes calculated with different methodologies.

Enzyme	Glide Score	Vina Score
UGM	−5.7	−9.3
PanK	−7.2	−9.0
MabA	−5.9	−7.5
InhA	−5.5	−7.1

Vina Score = binding energy calculated in AutoDock Vina. Docking score = binding energy estimation in Glide.

**Table 3:** Main interactions between the CG conformer of caespitate and essential amino acids for the activity of the four possible targets.

Protein	Nature of Interactions	Interaction with Amino Acid Residues
UGM	π–donor Hydrogen bond	Arg180
	π–alkyl	Tyr366
PanK	van der Waals	Phe254
	Hydrogen Bond	Tyr257
	π–alkyl	Tyr235
	van der Waals	Asn277
	π–π	Tyr182
MabA	van der Waals	Tyr153
	van der Waals	Ser140
	van der Waals	Lys157
	van der Waals	NAP
InhA	van der Waals	Arg254
	Hydrogen bond	NAD

**Table 4:** MM/GBSA binding free energies (kcal mol^−1^) calculated for UGM and PanK with the best poses with different methodologies.

Enzyme	MM/GBSA- ΔG-Bind Energy		MM/GBSA- ΔG-Bind-Coulomb		MM/GBSA- ΔG-Bind (NS)		MM/GBSA- ΔG-Bind (NS)-Coulomb	
	V	G	V	G	V	G	V	G
UGM	−53.2	−84.5	−8.9	−24.8	−61.7	−92.1	−6.9	−23.8
PanK	−56.5	−54.9	−4.5	−28.6	−60.4	−65.0	−4.49	−28.5

V = AutoDock Vina, G = Glide, MM/GBSA-ΔG-Bind = Complex − Receptor − Ligand and MMGBSA-ΔG-Bind (NS) = Complex − Receptor (from optimized complex) − Ligand (from optimized complex) = MMGBSA-ΔG-Bind − Receptor Strain − Ligand Strain. NS means no stain; this energy does not consider the conformational changes necessary for the formation of the complex.

**Table 5:** MM/GBSA docking score values, in kcal mol^−1^, obtained by the Glide program for each enzyme with conformers selected and calculated with different methodologies.

Enzyme	ΔG Bind Hbond		ΔG Bind Lipo		ΔG Bind Solv GB		ΔG Bind vdW	
	V	G	V	G	V	G	V	G
UGM	−1.9	−2.78	−36.1	−42.71	20.4	21.86	−31.3	−44.05
PanK	−0.9	−1.54	−22.6	−31.03	14.6	30.63	−22.9	−32.03

V = AutoDock Vina; G = Glide.

**Table 6:** Evaluation of the ADME and drug-like properties of caespitate conformers using QikProp and AdmetSAR.

Pharmacological Property	CG	CS	Pharmacological Property	CG	CS
MW ^a^	322.4	322.4	QplogBB ^f^	−1.2	−1.3
Donor HB ^b^	1.0	1.0	QplogS ^g^	−4.1	−4.0
Acceptor HB ^c^	4.3	4.3	Rule of Five	0.0	0.0
SASA ^d^	595.4	589.0	#metab ^h^	7.0	7.0
QPlogPo/w ^e^	3.2	3.2	%Human Oral Absortion ^i^	94.8	93.9
QPloghERG ^j^	−4.5	−4.7	AMES Toxicity ^k^	Non-toxic AMES/0.58	Non-toxic AMES/0.58

^a^ Molecular weight (acceptable range from <500); ^b^ hydrogen bond donor (acceptable range from ≤5); ^c^ hydrogen bond acceptor (acceptable range from ≤10); ^d^ total solvent-accessible surface area in square angstroms using a probe with a 1.4 radius (acceptable range from 300 to 1000); ^e^ predicted octanol/water partition coefficient (acceptable range from −2 to 6.5); ^f^ predicted blood/brain partition coefficient (acceptable range from −3 to 1.2); ^g^ predicted aqueous solubility, S in mol/dm^−3^ (acceptable range from −6.5 to 0.5); ^h^ number (#) of likely metabolic reactions (acceptable range from 1 to 8); ^i^ predicted human oral absorption on a 0 to 100% scale (<25% is poor and >80% is high); ^j^ predicted IC_50_ value for blockage of hERG K^+^ channels (concern below −5). ^k^ The predictive property can be classified as “toxic AMES” or “non-toxic AMES”. Values ranging from 0 to 1 quantify the certainty of the prediction, providing a measure of confidence in the assessment.

## Data Availability

Data is contained within the article and Supplementary Materials.

## Supplementary Materials

The following supporting information can be downloaded at: https://www.mdpi.com/article/10.3390/cimb46070387/s1, Table S1: Preservation of the IHBs and interactions between CS conformers and essential amino acids for the activity of the four enzymes proposed with docking in AutoDock Vina; Table S2: Affinity energies values (kcal mol^−1^) calculated for InhA and MabA enzyme substrates; Figure S1: Multiple sequence alignment of four selected targets: InhA (active site residues highlighted in pink), MabA (active site residues highlighted in yellow), PanK (active site residues highlighted in green), and UGM (active site residues highlighted in blue); Figure S2: Structural alignment of the four selected targets. The four proteins are represented in gray ribbons and the active sites in surface rendering, InhA orange, MabA blue, UGM ice blue, and PanK yellow. (A) InhA–MabA, (B) InhA–UGM, (C) InhA–PanK (D) PanK–MabA, (E) PanK–InhA, and (F) UGM–MabA; Figure S3: (A) Graphical 3D representation of the main interactions in the UGM–CG complex after semiflexible docking simulation in Glide. (B) Graphical 3D representation of the main interactions in the UGM–CG complex after semiflexible docking simulation in AutoDock Vina; Figure S4: (A) Graphical 3D representation of the main interactions in the PanK–CG complex after semiflexible docking simulation in Glide. (B) Graphical 3D representation of the main interactions in the PanK–CG complex after semiflexible docking simulation in AutoDock Vina; Figure S5: (A) Graphical 3D representation of the main interactions in the MabA–CG complex after semiflexible docking simulation in Glide. (B) Graphical 3D representation of the main interactions in the MabA–CG complex after semiflexible docking simulation in AutoDock Vina; Figure S6: (A) Graphical 3D representation of the main interactions in the InhA–CG complex after semiflexible docking simulation in Glide. (B) Graphical 3D representation of the main interactions in the InhA–CG complex after semiflexible docking simulation in AutoDock Vina; Figure S7: (A) Graphical 3D representation of the main interactions in the UGM–CG complex after rigid docking simulation in Glide. (B) Graphical 3D representation of the main interactions in the UGM–CG complex after rigid docking simulation in AutoDock Vina; Figure S8: (A) Graphical 3D representation of the main interactions in the PanK–CG complex after rigid docking simulation in Glide. (B) Graphical 3D representation of the main interactions in the PanK–CG complex after rigid docking simulation in AutoDock Vina; Figure S9: (A) Graphical 3D representation of the main interactions in the MabA–CG complex after rigid docking simulation in Glide. (B) Graphical 3D representation of the main interactions in the MabA–CG complex after rigid docking simulation in AutoDock Vina; Figure S10: (A) Graphical 3D representation of the main interactions in the InhA–CG complex after rigid docking simulation in Glide. (B) Graphical 3D representation of the main interactions in the InhA–CG complex after rigid docking simulation in AutoDock Vina; Figure S11: (A) Graphical 3D representation of the main interactions in the UGM–CS complex after rigid docking simulation in Glide. (B) Graphical 3D representation of the main interactions in the UGM–CS complex after rigid docking simulation in AutoDock Vina; Figure S12: (A) Graphical 3D representation of the main interactions in the PanK–CS complex after rigid docking simulation in Glide. (B) Graphical 3D representation of the main interactions in the PanK–CS complex after rigid docking simulation in AutoDock Vina; Figure S13: (A) Graphical 3D representation of the main interactions in the MabA–CS complex after rigid docking simulation in Glide. (B) Graphical 3D representation of the main interactions in the MabA–CS complex after rigid docking simulation in AutoDock Vina; Figure S14: (A) Graphical 3D representation of the main interactions in the InhA–CS complex after rigid docking simulation in Glide. (B) Graphical 3D representation of the main interactions in the InhA–CS complex after rigid docking simulation in AutoDock Vina.
