# Supplementary material for: In Silico Approach: Anti-Tuberculosis Activity of Caespitate in the H37Rv Strain
Source: Curr Issues Mol Biol. 2024 Jun 27;46(7):6489–507. doi: 10.3390/cimb46070387 (PMC11275643; doi:10.3390/cimb46070387)
Supplement: Supplementary file 1 [file cimb-46-00387-s001.zip › cimb-3015173-supplementary.pdf]

Supplementary Materials

In Silico Approach: Anti-tuberculosis Activity of Caespitate in H37Rv Strain.

Andrea Moreno-Ceballos[1], Norma A. Caballero[2,*], María Eugenia Castro[3], Jose Manuel Perez-Aguilar[1], Liliana Mammino[4], Francisco J. Melendez[1,*]

[1]Lab. de Química Teórica, Centro de Investigación, Depto. de Fisicoquímica, Facultad de Ciencias Químicas, Benemérita Universidad Autónoma de Puebla, Edif. FCQ10, 22 Sur y San Claudio, Ciudad Universitaria, Col. San Manuel, C.P 72570. Puebla, Puebla, México; andrea.morenoce@alumno.buap.mx; jmanuel.perez@correo.buap.mx; francisco.melendez@correo.buap.mx
[2]Facultad de Ciencias Biológicas, Benemérita Universidad Autónoma de Puebla, Edif. BIO1, 22 Sur y San Claudio, Ciudad Universitaria, Col. San Manuel, C.P 72570. Puebla, Puebla, México; norma.caballero@correo.buap.mx
[3]Centro de Química, Instituto de Ciencias, Benemérita Universidad Autónoma de Puebla, Complejo de Ciencias, ICUAP, Edif. IC8, 22 Sur y San Claudio, Ciudad Universitaria, Col. San Manuel, C.P 72570. Puebla, Puebla, México; mareug.castro@correo.buap.mx
[4]School of Mathematical and Natural Science, University of Venda, Thohoyandou 0950, South Africa; sasdestria@yahoo.com
*Correspondence: norma.caballero@correo.buap.mx; francisco.melendez@correo.buap.mx; Tel.: (2222295500 NAC ext 2819; FJM ext 2830)

Table S1. Preservation of the IHBs and interactions between CS conformers and essential amino acids for the activity of the four enzymes proposed with docking in AutoDock Vina.

**Complex**	**Preservation of IHBs**	**Interactions with amino residues**	**Nature of Interactions**
InhA-CS		Tyr158	Hydrogen bond
		Ala198	π-alkyl
	None	NAD1301	C-H bond and Unfavourable Donor-Donor
MabA-CS	None	Ser140	Hydrogen bond
		Tyr153	Hydrogen bond
		Lys157	Hydrogen bond
		NAP1249	C-H bond and Unfavourable Donor-Donor
UGM-CS	None	Arg180	Hydrogen bond and alkyl
		FAD401	π-σ
PanK-CS	2nd IHB	Tyr182	Hydrogen bond and π-alkyl
		Phe254	π-σ and π-alkyl
		Asn277	Hydrogen bond
		Tyr235	Hydrogen bond

Table S2. Affinity energies values (kcal mol^{-1}) calculated for InhA and MabA enzyme substrates.

**Enzyme**	**Substrate**	**Binding affinity**
InhA	THT	−9.5
UGM	UDP	−13.6

CLUSTAL O(1.2.4) multiple sequence alignment

```
sp|P9WPA7|COAA_MYCTU     ------------------------------------------------------MSR---     3
sp|P9WIQ1|GLF_MYCTU      --------MQPMTARFDLFVVGS---GFFGLTIAERVATQLDKRVLVLERRP----HIGG   45
sp|P9WGT3|MABA_MYCTU     MTATATEGAKPPFVSRSVLVTGGN--RGIGLAIAQRLAADGHKVAVTH--------R-GS   49
sp|P9WGR1|INHA_MYCTU     MTG--------LLDGKRILVSGIITDSSIAFHIARVAQEQGAQLVLTGFDRLRLIQRITD   52
                                           :

sp|P9WPA7|COAA_MYCTU     --LSEPSP--YVEF---------DRRQWRALRMSTPLALTEEE---------LVGLRG   39
sp|P9WIQ1|GLF_MYCTU      NAYSEAEPQTGIEVHKYGAHLFHTSNKRVWDYVRQFTDFTDYRHRVFAMHNGQAYQFPMG   105
sp|P9WGT3|MABA_MYCTU     G-APKGLF--GVECDVTDSDAVDRAFTAVEEH-------QGPVEV---------LVSNAG 90
sp|P9WGR1|INHA_MYCTU     R-LPAKAP--LLELDVQNEEHLASLAGRVTEAIGAGNKLDGVVHS-------IGFMPQTG   102
                              :*               .        *

sp|P9WPA7|COAA_MYCTU     LGEQIDLLEVEEVYLPLARLIHLQVAARQRLF------AATAEFLGEPQQNPDRPVPFII   93
sp|P9WIQ1|GLF_MYCTU      LGLVSQFFGKYFTPEQARQLIAEQ-AAEIDTADAQNLEEKAISLIGRP-----LYEAFVK   159
sp|P9WGT3|MABA_MYCTU     LSAD-AFLMRM-TEEKFEKVINANLTGAFRVA-QRASRSMQRNKFGRM-----IFIGSVS   142
sp|P9WGR1|INHA_MYCTU     MGIN-PFFDA-----PYADV-----SKGIHIS-AYSYASMAKALLPIM-----NPGGSIV   145
                     :.  ::     :  :           :       :

sp|P9WPA7|COAA_MYCTU     GVAGSVAVGKSTTARVLQALLARWDHHPRVDLVTTDGFLYPNAELQRRNLMHRKGFPESY   153
sp|P9WIQ1|GLF_MYCTU      G---------------YTAKQWQTDPKELPA---ANITR--------LPVRYTFDNRY   191
sp|P9WGT3|MABA_MYCTU     G-----SWG---------IGNQANYAASKAGVIGMARSIAR--------ELSKANVTANV   180
sp|P9WGR1|INHA_MYCTU     GMDFDPSRA---------MPAYNWMTVAKSALESVNRFVAR--------EAGKYGVRSNL   188
                 *               :    :      : .

sp|P9WPA7|COAA_MYCTU     -----NRRALMRFV----------------------TSVKSGSDYACAPVYSHLHYD   183
sp|P9WIQ1|GLF_MYCTU      FS---------DTYEGLPTD--------GYTAWLQNMAADHRI---------EVRLNTD   224
sp|P9WGT3|MABA_MYCTU     VAPGYIDTDMTRAL-----DE----RI---QQGALQFIPAK-RVGTPAEVAGVVSFLASE   227
sp|P9WGR1|INHA_MYCTU     VAAGPIRTLAMSAIVGGALGEEAGAQIQLLEEGWDQRAPIGWNMKDATPVAKTVCALLSD   248
                                 * .
sp|P9WPA7|COAA_MYCTU     IIPGAEQVVRHPDILILEGLNVLQTGPTLMVSDLFDFSLYVDARIEDI------EQWYVS   237
sp|P9WIQ1|GLF_MYCTU      WFDVRGQLRPG-----------SPAAPVVYTGPLDRYFDYAEGRLGWRTLDFEVEVLPIG   273
sp|P9WGT3|MABA_MYCTU     DASYI---------------------------SGAVIPVDGGMGMGH---------- 247
sp|P9WGR1|INHA_MYCTU     WLPAT---------------------------TGDIIYADGGAHTQLL---------   269
                        .:.

sp|P9WPA7|COAA_MYCTU     RFLAMRT---TAFADPESHFHHYAAFSDSQAV-----VAAREI----------WRTINR-   278
sp|P9WIQ1|GLF_MYCTU      DFQGTAVMNYNDLDVPYTRIHEFRHFHPERDYPTDKTVIMREYSRFAEDDDEPYYPINTE   333
sp|P9WGT3|MABA_MYCTU     ------------------------------------------------------- 247
sp|P9WGR1|INHA_MYCTU     -------------------------------------------------------   269

sp|P9WPA7|COAA_MYCTU     ----------------------------------------PNLVENILPTRPRATLV   295
sp|P9WIQ1|GLF_MYCTU      ADRALLATYRARAKSETASSKVLFGGRLGTYQYLDMHMAIASALNMYDNVLAPHLRDGVP   393
sp|P9WGT3|MABA_MYCTU     ------------------------------------------------------- 247
sp|P9WGR1|INHA_MYCTU     -------------------------------------------------------   269

sp|P9WPA7|COAA_MYCTU     LRKDADHSINRLRLRKL        312
sp|P9WIQ1|GLF_MYCTU      LLQDGA-----------399
sp|P9WGT3|MABA_MYCTU     ----------------   247
sp|P9WGR1|INHA_MYCTU     ----------------   269
```

Figure S1. Multiple Sequence Alignment of Four Selected Targets: InhA (active site residues highlighted in pink), MabA (active site residues highlighted in yellow), PanK (active site residues highlighted in green), and UGM (active site residues highlighted in blue).

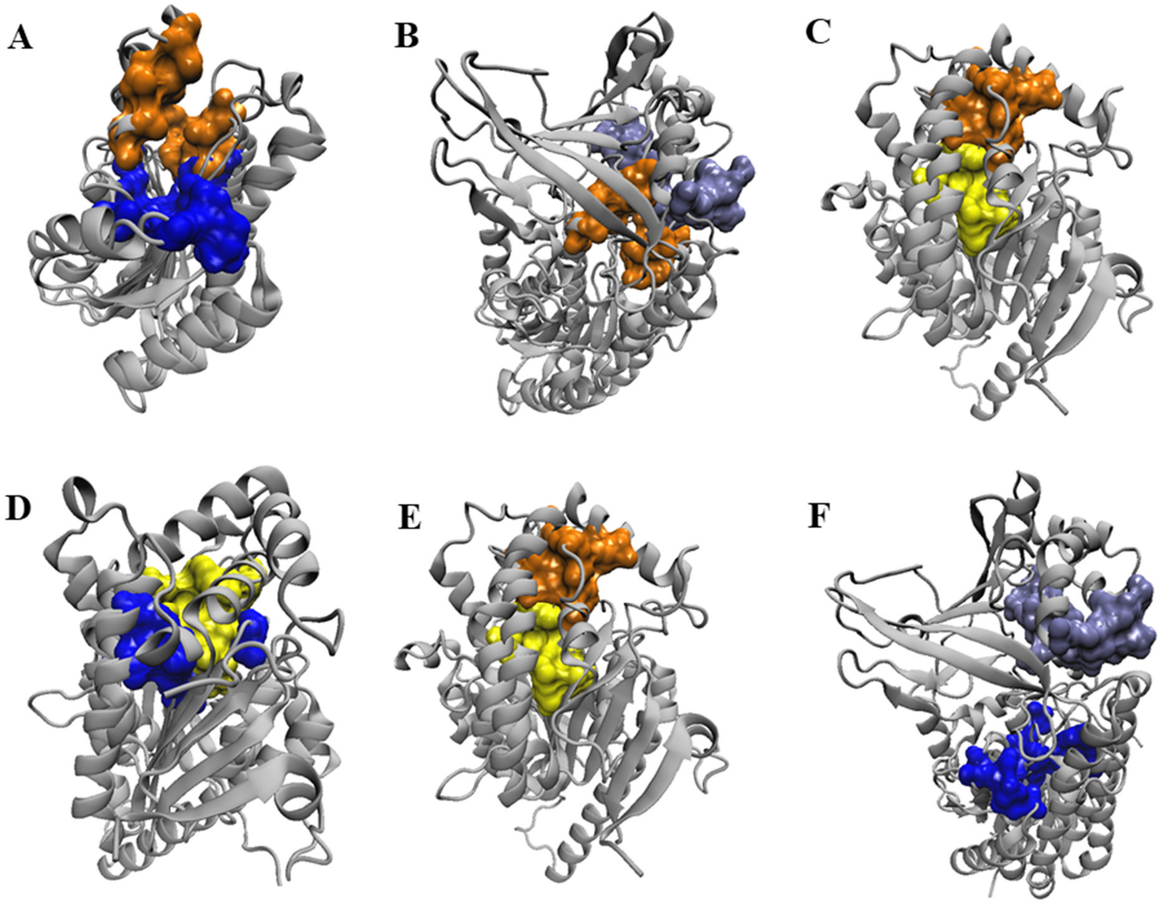

Figure S2. Structural alignment of the four selected targets. The four proteins are represented in grey ribbons and the active sites in surface rendering, InhA orange, MabA blue UGM iceblue, and PanK yellow. A. InhA-MabA, B. InhA-UGM, C. InhA-PanK D. PanK-MabA, E. PanK-InhA, F. UGM-MabA.

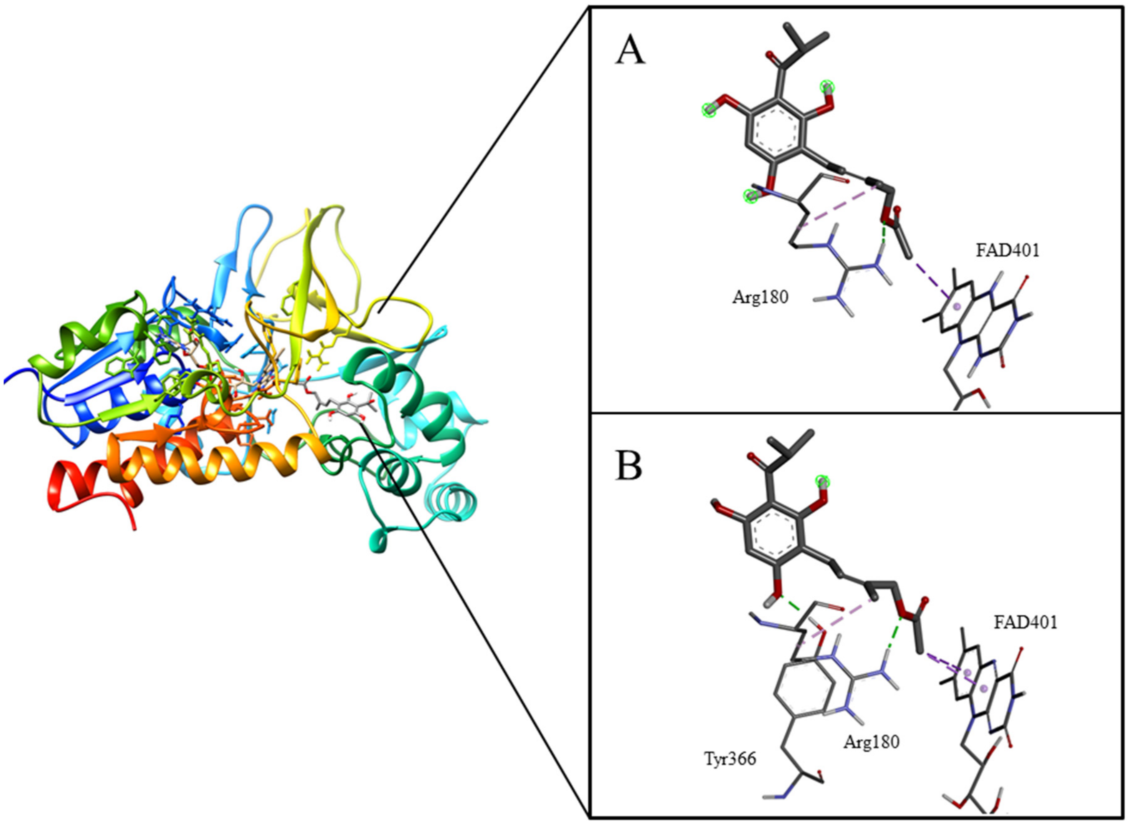

Figure S3 A. Graphical 3D representation of the main interactions in UGM-CG complex after semiflexible docking simulation in Glide B. Graphical 3D representation of the main interactions in UGM-CG complex after semiflexible docking simulation in AutoDock Vina.

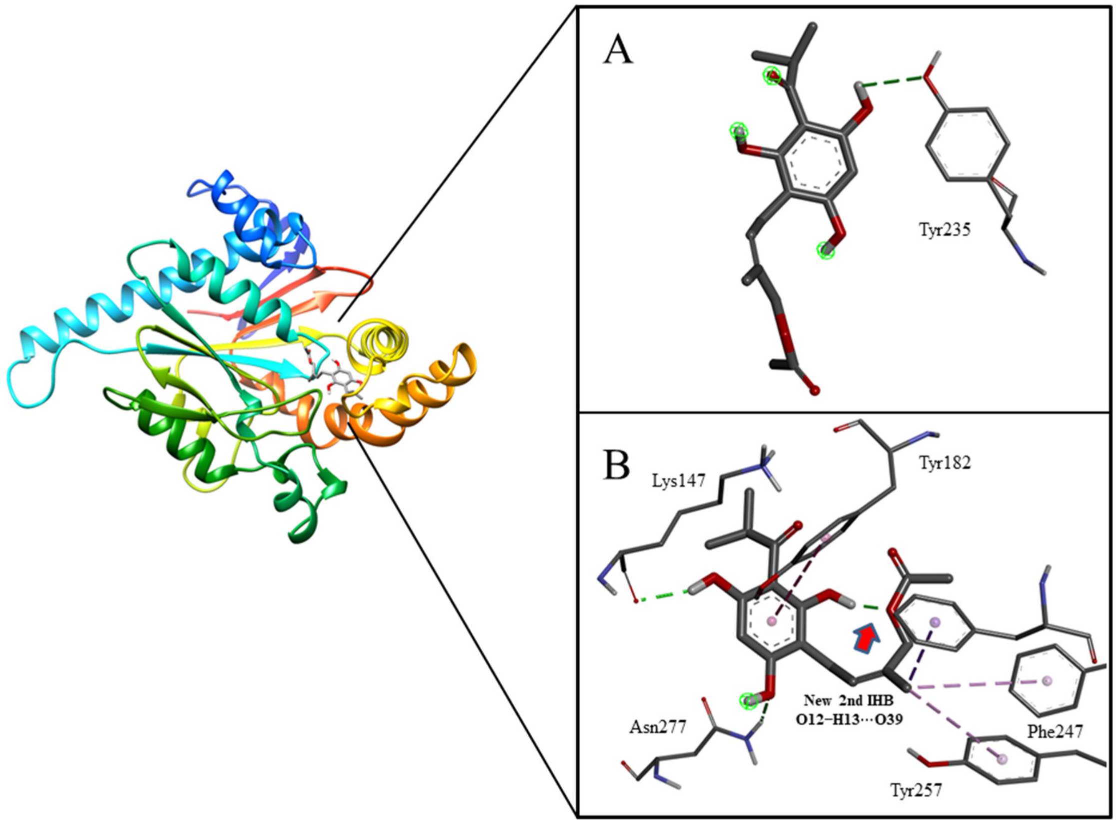

Figure S4. A. Graphical 3D representation of the main interactions in PanK-CG complex after semiflexible docking simulation in Glide B. Graphical 3D representation of the main interactions in PanK-CG complex after semiflexible docking simulation in AutoDock Vina.

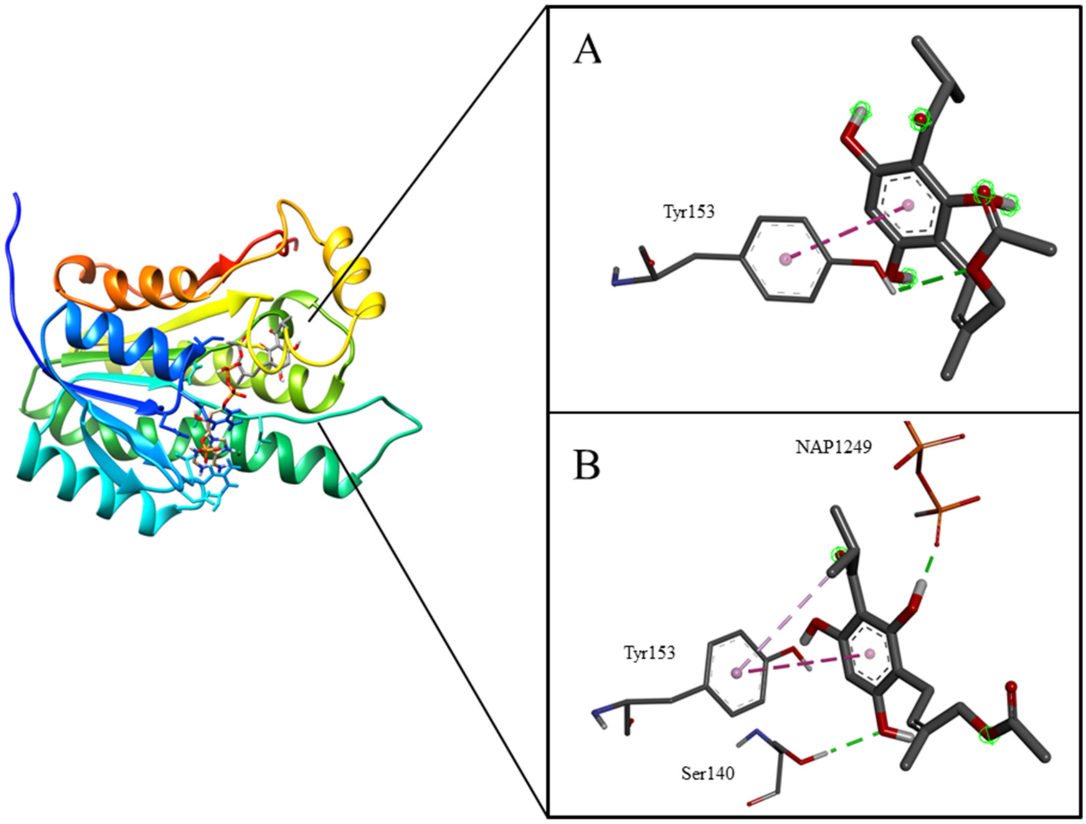

Figure S5. A. Graphical 3D representation of the main interactions in MabA-CG complex after semiflexible docking simulation in Glide B. Graphical 3D representation of the main interactions in MabA-CG complex after semiflexible docking simulation in AutoDock Vina.

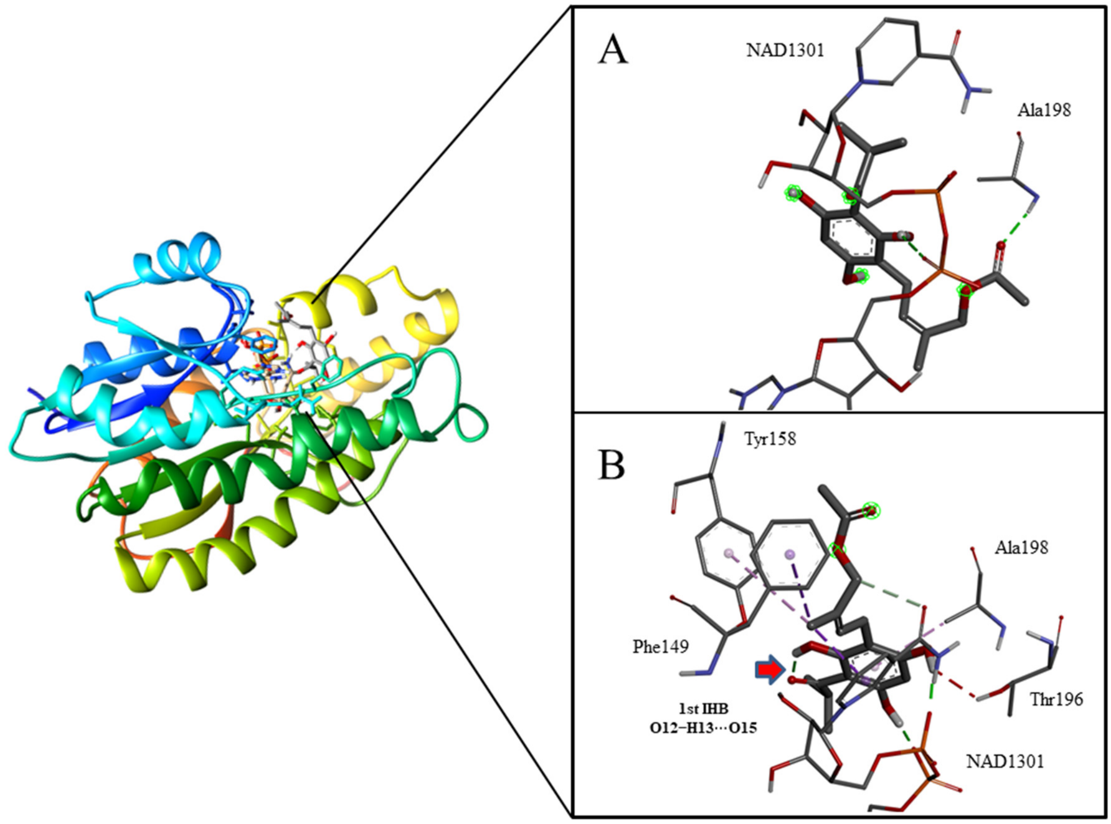

Figure S6. A. Graphical 3D representation of the main interactions in InhA-CG complex after semiflexible docking simulation in Glide B. Graphical 3D representation of the main interactions in InhA-CG complex after semiflexible docking simulation in AutoDock Vina.

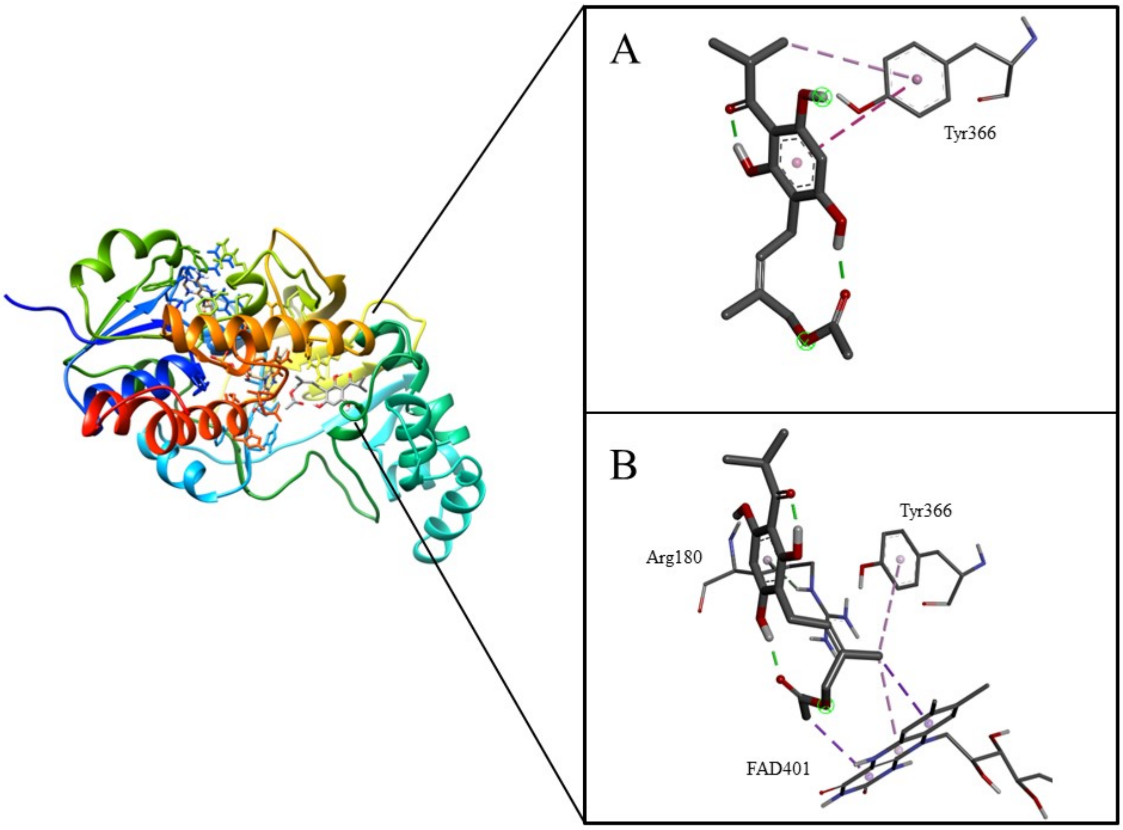

Figure S7. A. Graphical 3D representation of the main interactions in UGM-CG complex after rigid docking simulation in Glide B. Graphical 3D representation of the main interactions in UGM-CG complex after rigid docking simulation in AutoDock Vina.

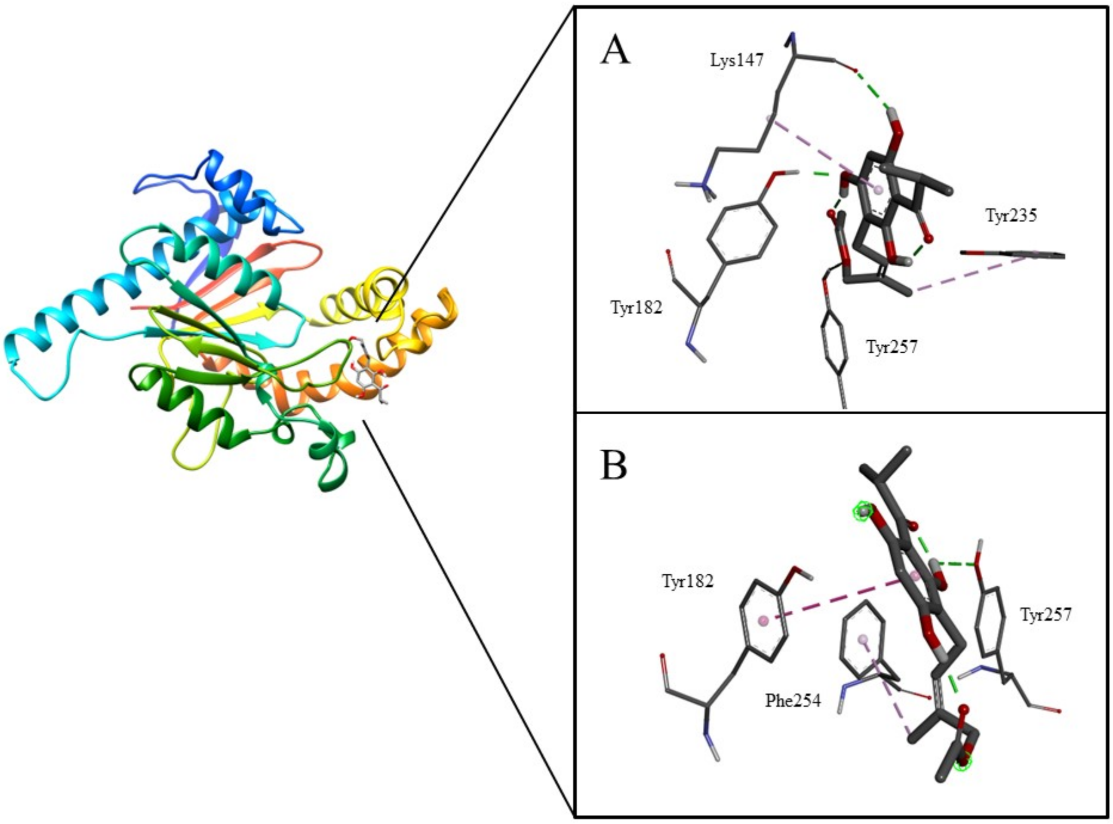

Figure S8. A. Graphical 3D representation of the main interactions in PanK-CG complex after rigid docking simulation in Glide B. Graphical 3D representation of the main interactions in PanK-CG complex after rigid docking simulation in AutoDock Vina.

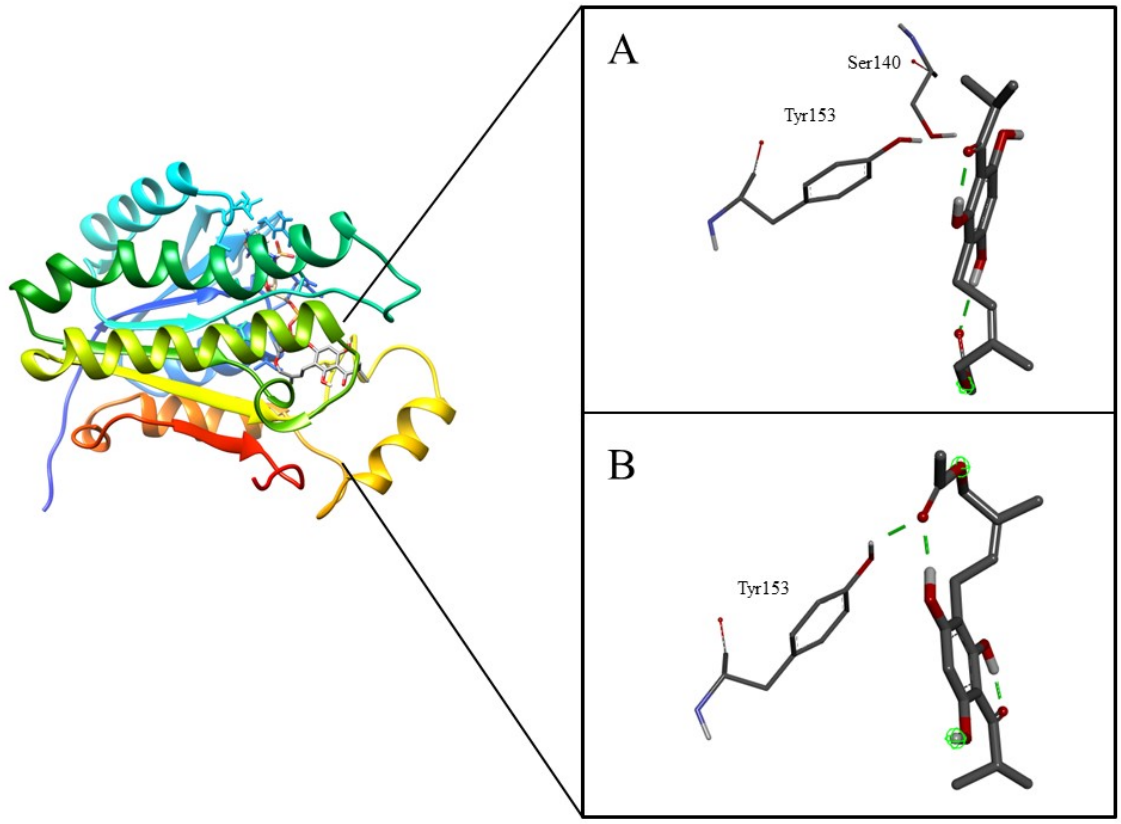

Figure S9. A. Graphical 3D representation of the main interactions in MabA-CG complex after rigid docking simulation in Glide B. Graphical 3D representation of the main interactions in MabA-CG complex after rigid docking simulation in AutoDock Vina.

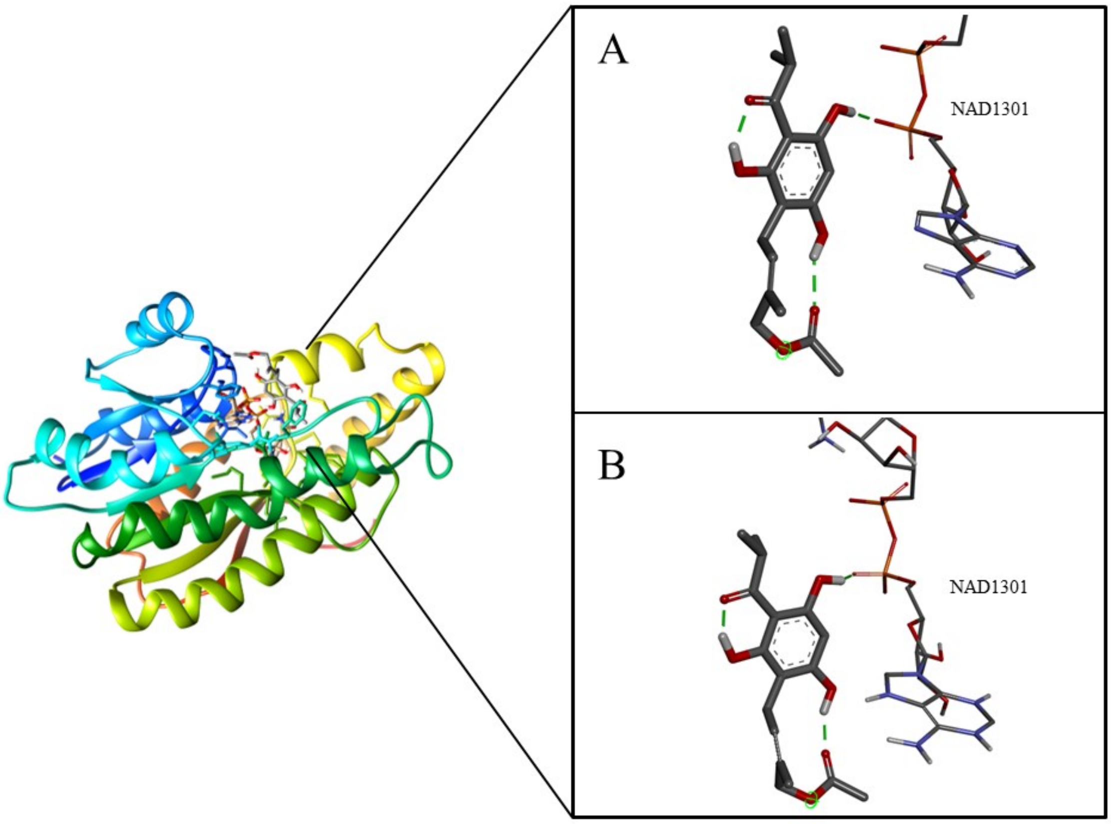

Figure S10. A. Graphical 3D representation of the main interactions in InhA-CG complex after rigid docking simulation in Glide B. Graphical 3D representation of the main interactions in InhA-CG complex after rigid docking simulation in AutoDock Vina.

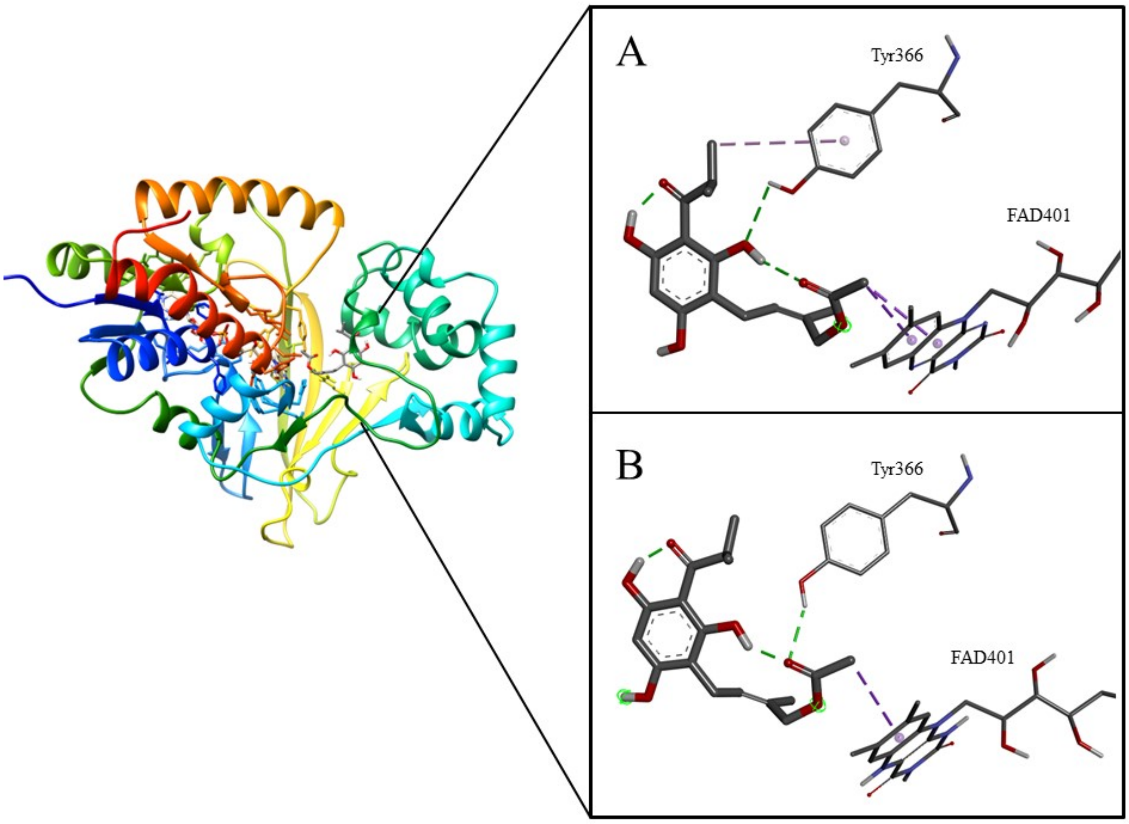

Figure S11. A. Graphical 3D representation of the main interactions in UGM-CS complex after rigid docking simulation in Glide B. Graphical 3D representation of the main interactions in UGM-CS complex after rigid docking simulation in AutoDock Vina.

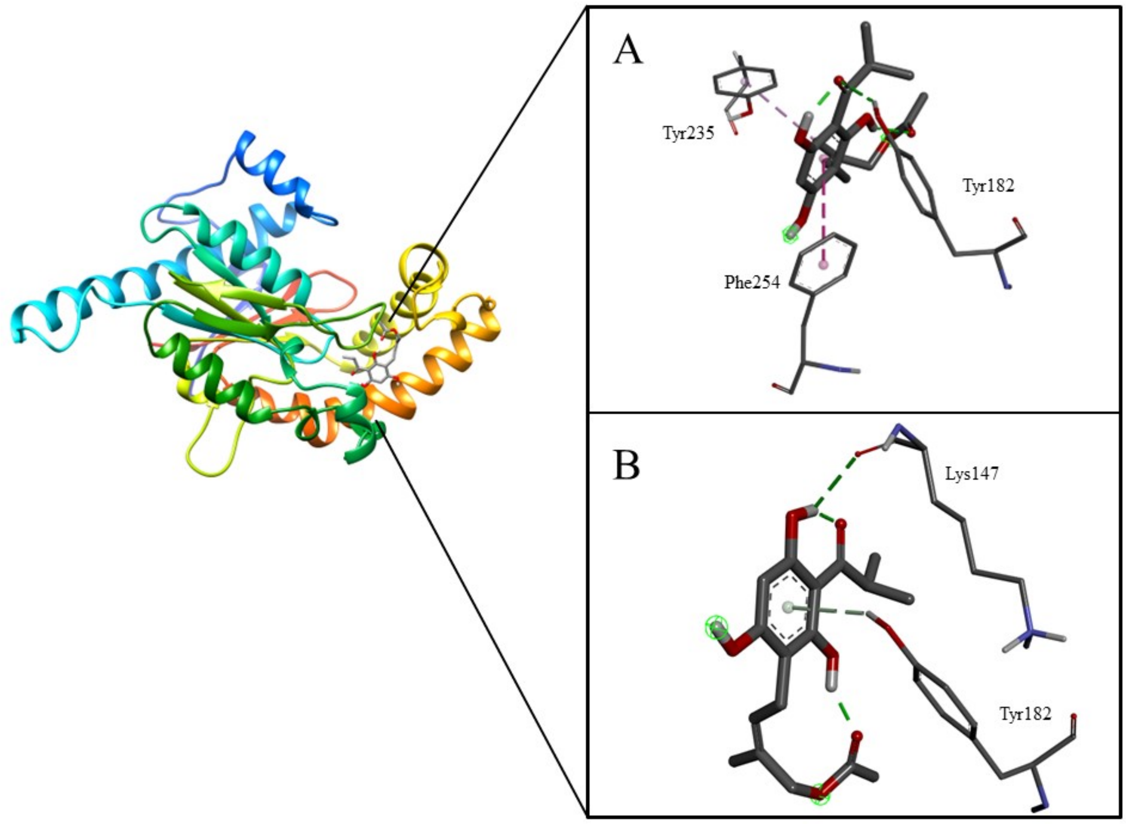

Figure S12. A. Graphical 3D representation of the main interactions in PanK-CS complex after rigid docking simulation in Glide B. Graphical 3D representation of the main interactions in PanK-CS complex after rigid docking simulation in AutoDock Vina.

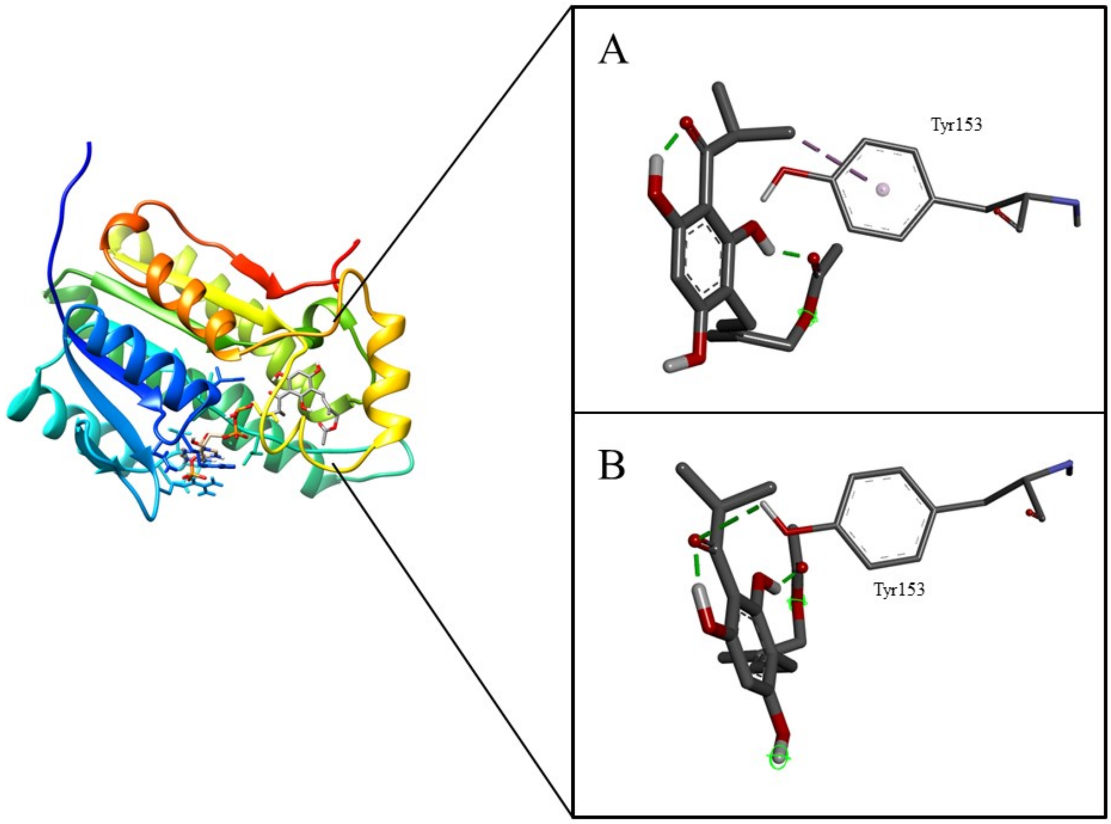

Figure S13**.** A. Graphical 3D representation of the main interactions in MabA-CS complex after rigid docking simulation in Glide B. Graphical 3D representation of the main interactions in MabA-CS complex after rigid docking simulation in AutoDock Vina.

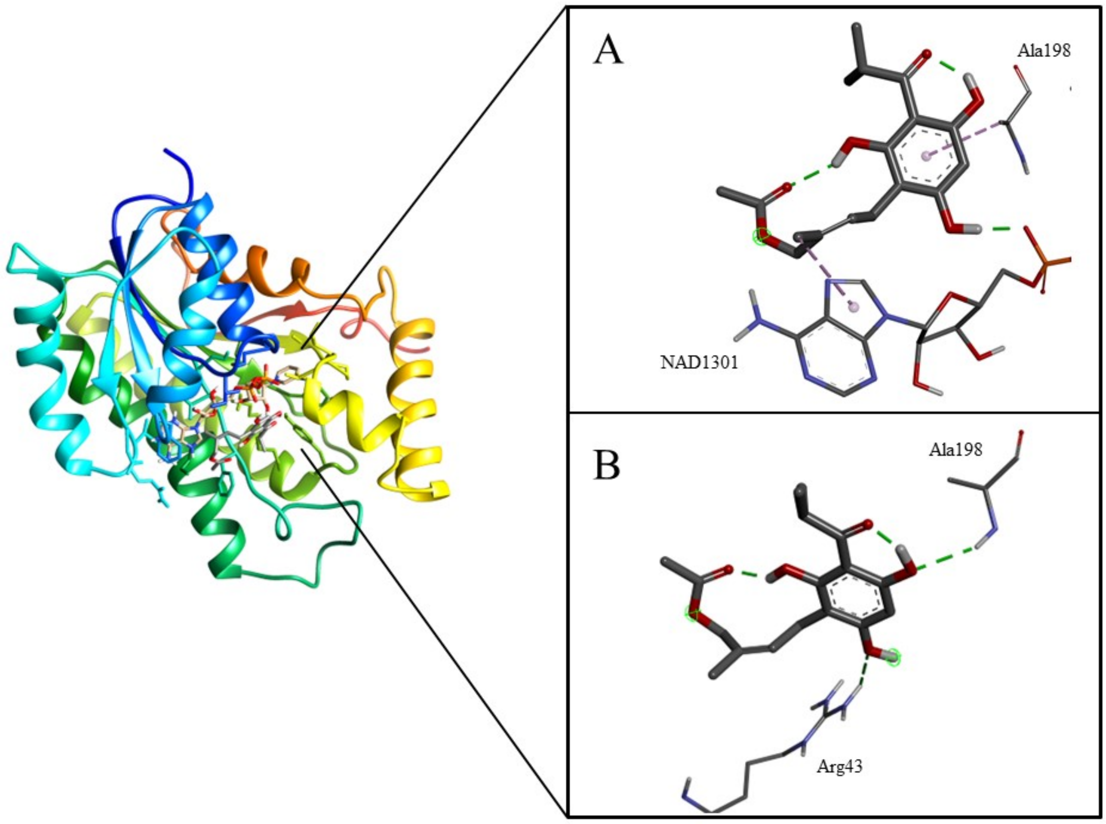

Figure S14. A. Graphical 3D representation of the main interactions in InhA-CS complex after rigid docking simulation in Glide B. Graphical 3D representation of the main interactions in InhA-CS complex after rigid docking simulation in AutoDock Vina.
